# excluderanges: exclusion sets for T2T-CHM13, GRCm39, and other genome assemblies

**DOI:** 10.1093/bioinformatics/btad198

**Published:** 2023-04-17

**Authors:** Jonathan D Ogata, Wancen Mu, Eric S Davis, Bingjie Xue, J Chuck Harrell, Nathan C Sheffield, Douglas H Phanstiel, Michael I Love, Mikhail G Dozmorov

**Affiliations:** Department of Biostatistics, Virginia Commonwealth University, Richmond, VA 23298, United States; Department of Biostatistics, University of North Carolina-Chapel Hill, Chapel Hill, NC 27514, United States; Curriculum in Bioinformatics and Computational Biology, University of North Carolina at Chapel Hill, Chapel Hill, NC 27599, United States; Center for Public Health Genomics, University of Virginia, Charlottesville, VA 22908, United States; Department of Pathology, Virginia Commonwealth University, Richmond, VA 23284, United States; Massey Cancer Center, Virginia Commonwealth University, Richmond, VA 23220, United States; Center for Public Health Genomics, University of Virginia, Charlottesville, VA 22908, United States; Curriculum in Bioinformatics and Computational Biology, University of North Carolina at Chapel Hill, Chapel Hill, NC 27599, United States; Thurston Arthritis Research Center, University of North Carolina at Chapel Hill, Chapel Hill, NC 27599, United States; Department of Cell Biology and Physiology, University of North Carolina at Chapel Hill, Chapel Hill, NC 27599, United States; Lineberger Comprehensive Cancer Center, University of North Carolina at Chapel Hill, Chapel Hill, NC 27599, United States; Curriculum in Genetics and Molecular Biology, University of North Carolina at Chapel Hill, Chapel Hill, NC 27599, United States; Department of Biostatistics, University of North Carolina-Chapel Hill, Chapel Hill, NC 27514, United States; Department of Genetics, University of North Carolina-Chapel Hill, Chapel Hill, NC 27514, United States; Department of Biostatistics, Virginia Commonwealth University, Richmond, VA 23298, United States; Department of Pathology, Virginia Commonwealth University, Richmond, VA 23284, United States

## Abstract

**Summary:**

Exclusion regions are sections of reference genomes with abnormal pileups of short sequencing reads. Removing reads overlapping them improves biological signal, and these benefits are most pronounced in differential analysis settings. Several labs created exclusion region sets, available primarily through ENCODE and Github. However, the variety of exclusion sets creates uncertainty which sets to use. Furthermore, gap regions (e.g. centromeres, telomeres, short arms) create additional considerations in generating exclusion sets. We generated exclusion sets for the latest human T2T-CHM13 and mouse GRCm39 genomes and systematically assembled and annotated these and other sets in the *excluderanges* R/Bioconductor data package, also accessible via the BEDbase.org API. The package provides unified access to 82 GenomicRanges objects covering six organisms, multiple genome assemblies, and types of exclusion regions. For human hg38 genome assembly, we recommend *hg38.Kundaje.GRCh38_unified_blacklist* as the most well-curated and annotated, and sets generated by the Blacklist tool for other organisms.

**Availability and implementation:**

https://bioconductor.org/packages/excluderanges/. Package website: https://dozmorovlab.github.io/excluderanges/.

## 1 Introduction

Up to 87% of sequencing reads generated by chromatin targeting technologies (e.g. ChIP-seq) can map to a reference genome in distinct clusters (aka high-signal pileups) (https://docs.google.com/spreadsheets/d/1G4SkqUMiGcUlvR6homc7RW33nSOf4mS9QYJifsd4qo0; https://sites.google.com/site/anshulkundaje/projects/blacklists) (Amemiya et al. 2019). These pileups frequently occur in regions near assembly gaps, copy number-high regions, and in low-complexity regions (Pickrell et al. 2011; Miga et al. 2015). Removing reads overlapping those regions, referred hereafter as exclusion sets, improves normalization of the signal between samples, correlation between replicates, and increases accuracy of both peak calling and differential ChIP-seq analysis (Kharchenko et al. 2008; Carroll et al. 2014; Wimberley and Heber 2020). Therefore, standardized availability of those exclusion sets is critical for improving reproducibility and quality of bioinformatics analyses.

Finding and choosing an exclusion set can be a non-trivial task. The ENCODE project returns 94 hits using the “exclusion” search term (as of 11 August 2022) (https://www.encodeproject.org/search/?searchTerm=exclusion), most of them having minimal annotation and unknown curation methods. These sets are available for human and mouse genome assemblies; however, the ENCODE project lacks exclusion sets for the latest Telomere-to-Telomere (T2T-CHM13) human and Genome Reference Consortium Mouse Build 39 (GRCm39/mm39) mouse assemblies. Converting exclusion set coordinates between genomic assemblies using liftOver is not advisable since new artifact–prone regions are added and others are lost due to closed gaps (Amemiya et al. 2019); therefore, exclusion sets should be generated and used for their respective genome assemblies. Furthermore, exclusion regions have been observed in genomes of other species and many exclusion sets for model organisms remain unpublished and scattered across GitHub repositories. We curated a collection of exclusion sets for six model organisms and 12 genome assemblies, including the newly generated T2T and mm39 exclusion sets. We included two other types of potentially problematic regions: University of California Santa Cruz (UCSC)-annotated gap sets, e.g. centromere, telomere, short arm, and nuclear mitochondrial (NUMT) sets containing mitochondrial sequences present in the nuclear genome (Qu et al. 2008). We assemble a total of 82 uniformly processed and annotated exclusion sets in the *excluderanges* R/Bioconductor data package and provide API access via BEDbase.org.

## 2 Implementation

**Figure 1.**
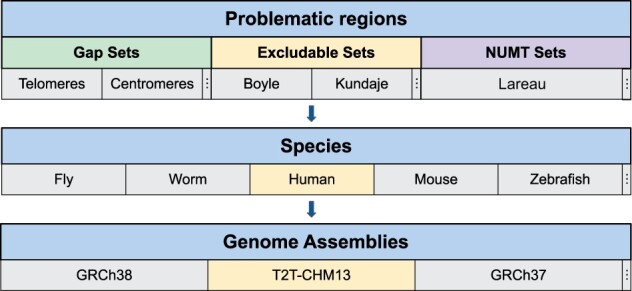
Schematic overview of the *excluderanges* package. Data for each type of problematic region [exclusion sets, gaps, nuclear mitochondrial (NUMT) sets] were obtained from public sources for each model organism and the corresponding genome assemblies. Exclusion sets for T2T-CHM13 and GRCm39 genome assemblies were *de novo* generated. Three vertical dots indicate more categories in the corresponding section

An overview of the *excluderanges* data is shown in Fig. 1. To create this resource, we performed a systematic internet and literature search. The ENCODE project was the largest source of exclusion sets for human (11 sets) and mouse (6 sets) organisms, covering hg19, hg38, mm9, and mm10 genome assemblies. We also obtained exclusion sets generated by the Blacklist (Amemiya et al. 2019) and PeakPass (Wimberley and Heber 2020) software. Additionally, we obtained exclusion sets for *Caenorhabditis elegans* (*C. elegans,* ce10 and ce11 genome assemblies), *Drosophila melanogaster* (*D. melanogaster,* dm3 and dm6), *Danio rerio* (*D. rerio,* danRer10), and *A. thaliana* (TAIR10). Using the Blacklist software, we generated exclusion sets for the latest Telomere-to-Telomere (T2T-CHM13) human and Genome Reference Consortium Mouse Build 39 (GRCm39/mm39) mouse assemblies (Table 1, Supplementary Table S1).

**Table 1.** Characteristics of recommended exclusion sets for human and mouse genome assemblies.^a^

Name	Assembly	Number of regions	Width, min/median/max, bp	Percent of the genome, %	Year last updated
*T2T.excluderanges*	T2T	2066	1001/9701/25 738 901	8.358	2022
*hg38.Kundaje.GRCh38_unified_Excludable* ^b^	hg38	910	19/384/5 407 756	2.317	2020
*hg38.Boyle.hg38-Excludable.v2*	hg38	636	1200/10 150/30 590 100	7.355	2018
*hg38.Wimberley.peakPass60Perc_sorted* ^c^	hg38	5078	1000/2000/1 852 000	2.387	2021
*hg19.Boyle.hg19-Excludable.v2*	hg19	834	1100/9350/30 590 100	8.882	2018
*mm39.excluderanges*	mm39	3147	1100/12 500/5 487 000	6.272	2022
*mm10.Boyle.mm10-Excludable.v2*	mm10	3435	1000/8100/50 585 400	8.768	2018

a Unless specified otherwise, exclusion sets were defined by the Boyle-Lab/Blacklist software. The complete list is provided in Supplementary Table S1.

b Defined as a combination of *hg38.Lareau.hg38_peaks*, *hg38.Boyle.hg38-Excludable.v2*, and *hg38.Wimberley.peakPass60Perc_sorted*, followed by manual curation, https://www.encodeproject.org/files/ENCFF356LFX/.

c Defined by the PeakPass software, https://github.com/ewimberley/peakPass/raw/main/excludedlists/.

Mitochondrial DNA sequences (mtDNA, 100–600K mitochondria per human cell) transferred to the nucleus give rise to the so-called mitochondrial DNA sequences in the nuclear genome (NUMTs). These sequences are found in genomes of various species (Qu et al. 2008), suggesting NUMTs may be a pervasive phenomenon. In the settings of DNA/chromatin sequencing (e.g. ATAC-seq), up to 80% of mitochondrial sequencing reads (Montefiori et al. 2017) may pile up in the NUMT sequences. Similar to exclusion sets, genomic regions highly homologous to mtDNA can be masked to improve biological signal. The reference human NUMT sequences have been available in the UCSC genome browser for hg18 [RHNumtS.2 database (Simone et al. 2011)] and lifted over to hg19 human genome assembly. Similarly, mouse NUMTs [RMNumtS database (Calabrese et al. 2012)] are available for the mm9 mouse genome assembly. However, recent human, mouse, and other organism genome assemblies lack NUMTs annotations in the UCSC database. We collected NUMT sets for more recent human and mouse genome assemblies, including hg38, T2T-CHM13, mm10, generated by Caleb Lareau in the mitoblacklist GitHub repository (https://github.com/caleblareau/mitoblacklist).

Gaps in the genome represent another type of problematic regions. These include centromere and telomere sequences, short arms, gaps from large heterochromatin blocks, etc. While some are present in genome assemblies of most organisms (centromeres, telomeres, short arms, covering 2.47% ± 1.64, 0.01% ± 0.01, and 15.39% ± 3.66 of hg38 chromosomes, respectively), many are assembly-specific (e.g. gaps between clones, contigs, scaffolds in hg19 and hg38 assemblies). Gap data are available from the UCSC Genome Browser database or UCSC-hosted data hubs. The T2T-CHM13 assembly lacks assembly-specific gaps by the definition of telomere-to-telomere sequencing (Nurk et al. 2022); however, coordinates of centromeres and telomeres are available from the CHM13 GitHub repository (https://github.com/marbl/CHM13). Additionally, we obtained T2T peri/centromeric satellite annotations, known to be associated with constitutive heterochromatin and span sites involved in kinetochore assembly or sequences epigenetically marked as centromeres (Yunis and Yasmineh 1971). We also included the rDNA gap regions and regions unique to T2T-CHM13 v2.0 as compared with GRCh38/hg38 and GRCh37/hg19 assemblies under the rationale that alignments within these previously problematic regions might warrant extra attention. We characterized hg38 exclusion sets for overlap with gap regions and found that *hg38.Kundaje.GRCh38_unified_Excludable*, *hg38.Boyle.hg38-Excludable.v2*, and *hg28.Wimberley.peakPass60Perc_sorted* cover 99.40%, 99.08%, and 59.60% of centromeric regions, respectively. Notably, relatively few large regions were responsible for these overlaps (e.g. 27 out of 910 in *hg38.Kundaje.GRCh38_unified_Excludable*). In contrast, over 60% of the *hg38.Nordin.CandRblacklist_hg38* exclusion set for the CUT&RUN technology overlapped centromeres on chromosomes 1 and 13. Only sets generated by the Blacklist software overlapped centromeres, telomeres, and short arms, and there results were consistent across organisms and genome assemblies (Supplementary Table S2). Given the distinct properties of gap regions and inconsistency of their presence in exclusion sets, the aforementioned NUMTs and gap sets may be combined with other exclusion sets.

The large number of exclusion sets (e.g. nine for hg38 human genome assemblies) creates uncertainty in which set to use for a given genome assembly. We annotated exclusion sets by their creation methods, date of last update, width distribution, percent of the genome covered, and other properties (Supplementary Table S1, BEDbase.org; example of BEDbase overview screen for hg38.Kundaje.GRCh38_unified_blacklist: http://bedbase.org/#/bedsplash/1a561729234c2844303a051b16f66656). Only sets generated by the Boyle’s lab Blacklist (Amemiya et al. 2019) or PeakPass by Eric Wimberley (Wimberley and Heber 2020) software had published methods. While methods for some sets may be inferred (e.g. the hg38 *Yeo.eCLIP_Excludableregions.hg38liftover* set may have been lifted over from hg19), we advise against using poorly annotated sets. We also characterized hg38 exclusion sets and found they vary dramatically in terms of number (12 052—38) and width (median 10 151—30 bp) (Supplementary Fig. S1A and B). We calculated Jaccard overlap between each pair of hg38 exclusion sets, $J\left(A,\;B\right)\;=\;\frac{\mathrm{width}\left(\cap_{A,B}\right)}{\mathrm{width}\left(\cup_{A,B}\right)}$. We found that *hg38.Kundaje.GRCh38_unified_Excludable* had the best Jaccard overlap with other sets, followed by_hg38.Wimberley.peakPass60Perc_sorted_ and *hg38.Boyle.hg38-Excludable.v2* sets (Supplementary Fig. S1C). We additionally calculated overlap coefficient $C\left(A,\;B\right)=\frac{\mathrm{width}\left(\cap_{A,B}\right)}{\mathrm{Min}\left(\mathrm{width}\left(A\right),\mathrm{width}\left(B\right)\right)}$ to minimize the effect of set size differences. We similarly found Kindaje-generated sets showing the best overlap with other sets, followed by *hg38.Boyle.hg38-Excludable.v2*. We also observed *hg38.Wold.hg38mitoExcludable* and *hg38.Lareau.hg38.full.Excludable* sets overlapping *hg38.Kundaje.GRCh38_unified_Excludable*, suggesting it contains NUMTs (Supplementary Fig. S1D). Because of its agreement with other sets, we recommend *hg38.Kundaje.GRCh38_unified_Excludable* set and list other recommended sets Table 1.

## 3 Discussion

Limited annotation remains the main problem when selecting exclusion sets as it remains unclear which method and/or data were used. Examples include Wold’s lab-generated “mitoblack” sets for mm9 and mm10 assemblies. Their curation method is unknown, and the exact number (123 regions), width distribution, and other characteristics suggest that one may be a liftOver version of the other. Similarly, it remains unknown why Bernstein’s lab-generated “Mint_Blacklist” hg19 and hg38 exclusion sets have a very large number of regions (9,035 and 12 052, respectively) as compared with under 1000 regions for other exclusion sets. Additionally, hg19 and hg38 “full.blacklist” sets were generated by Caleb Lareau as a combination of NUMTs and unknown ENCODE exclusion sets, the source of which we were unable to infer. Given annotation shortcomings, we discourage creating a union of all exclusion sets and recommend using exclusion sets generated with published methods and, if relevant, combining them with gap sets.

Most annotated exclusion sets were created via Blacklist, a tool for detecting regions with abnormally high signal and/or low mappability (Amemiya et al. 2019). These genomic properties are commonly accepted as problematic; however, they may not be exhaustive. The Peakpass algorithm was developed to learn genomic properties associated with problematic regions using a random forest model (Wimberley and Heber 2020). It reported distance to nearest assembly gap or gene, and frequency of unique 4-mers or softmasked base pairs, as the most predictive of problematic regions. A limitation of Peakpass is that its extensive collection of Python, R, and bash scripts is poorly documented. A limitation of Blacklist, on the other hand, is computational resource requirements (64+ GB; CPU: 24+ cores, 3.4+ GHz/core) and disk storage (∼1 TB) due to a large number of required BAM files (hundreds). A recent preprint introduced the Greenscreen pipeline, a promising tool for identifying exclusion sets using as few as three ChIP-seq data. It reports a 99.9% overlap with a Blacklist-generated exclusion set, identical performance on ChIP-seq quality metrics but a smaller genome footprint (Klasfeld and Wagner 2022). We utilized Blacklist as the most well-known tool to generate exclusion sets for the T2T-CHM13 and GRCm39 genome assemblies. The aforementioned tools detect problematic regions in ChIP-seq data; however, they may be different in data generated by other technologies due to different biochemical procedures (Nordin et al. 2022). Additional collaborative efforts are needed to develop a consensus approach for defining well-documented exclusion sets.

## Supplementary Material

btad198_Supplementary_Data

## Data Availability

Data are available in a GitHub repository https://dozmorovlab.github.io/excluderanges/ and can be accessed via a DOI link https://doi.org/doi:10.18129/B9.bioc.excluderanges.

## References

[btad198-B1] Amemiya HM , Kundaje A, Boyle AP. The ENCODE blacklist: identification of problematic regions of the genome. Sci Rep 2019;9:9354. 31249361 10.1038/s41598-019-45839-z PMC6597582

[btad198-B2] Calabrese FM , Simone D, Attimonelli M. Primates and mouse NumtS in the UCSC genome browser. BMC Bioinformatics 2012;13(Suppl 4):S15. 10.1186/1471-2105-13-S4-S15 PMC3314570 22536961

[btad198-B3] Carroll TS , Liang Z, Salama R et al Impact of artifact removal on ChIP quality metrics in ChIP-seq and ChIP-exo data. Front Genet 2014;5:75. 24782889 10.3389/fgene.2014.00075 PMC3989762

[btad198-B4] Kharchenko PV , Tolstorukov MY, Park PJ. Design and analysis of ChIP-seq experiments for DNA-binding proteins. Nat Biotechnol 2008;26:1351–9. 19029915 10.1038/nbt.1508 PMC2597701

[btad198-B5] Klasfeld S , Wagner D. Greenscreen decreases type I errors and increases true peak detection in genomic datasets including ChIP-seq. *bioRxiv* 2022. 10.1101/2022.02.27.482177. PMC9709979 36124976

[btad198-B6] Miga KH , Eisenhart C, Kent WJ. Utilizing mapping targets of sequences underrepresented in the reference assembly to reduce false positive alignments. Nucleic Acids Res 2015;43:e133. 26163063 10.1093/nar/gkv671 PMC4787761

[btad198-B7] Montefiori L , Hernandez L, Zhang Z et al Reducing mitochondrial reads in ATAC-seq using CRISPR/Cas9. Sci Rep 2017;7:2451. 28550296 10.1038/s41598-017-02547-w PMC5446398

[btad198-B8] Nordin A , Zambanini G, Pagella P et al The CUT&RUN blacklist of problematic regions of the genome. *bioRxiv* 2022. 10.1101/2022.11.11.516118. PMC10416431 37563719

[btad198-B9] Nurk S , Koren S, Rhie A et al The complete sequence of a human genome. Science 2022;376:44–53. 35357919 10.1126/science.abj6987 PMC9186530

[btad198-B10] Pickrell JK , Gaffney DJ, Gilad Y et al False positive peaks in ChIP-seq and other sequencing-based functional assays caused by unannotated high copy number regions. Bioinformatics 2011;27:2144–6. 21690102 10.1093/bioinformatics/btr354 PMC3137225

[btad198-B11] Qu H , Ma F, Li Q. Comparative analysis of mitochondrial fragments transferred to the nucleus in vertebrate. J Genet Genomics 2008;35:485–90. 18721785 10.1016/S1673-8527(08)60066-1

[btad198-B12] Simone D , Calabrese FM, Lang M et al The reference human nuclear mitochondrial sequences compilation validated and implemented on the UCSC genome browser. BMC Genomics 2011;12:517. 22013967 10.1186/1471-2164-12-517 PMC3228558

[btad198-B13] Wimberley CE , Heber S. PeakPass: automating ChIP-seq blacklist creation. J Comput Biol 2020;27:259–68. 10.1089/cmb.2019.0295 31855064

[btad198-B14] Yunis JJ , Yasmineh WG. Heterochromatin, satellite DNA, and cell function. Structural DNA of eucaryotes may support and protect genes and aid in speciation. Science 1971;174:1200–9. 4943851 10.1126/science.174.4015.1200

