# Supplementary figures and images for: excluderanges: exclusion sets for T2T-CHM13, GRCm39, and other genome assemblies
Source: Bioinformatics. 2023 Apr 17;39(4):btad198. doi: 10.1093/bioinformatics/btad198 (PMC10126321; doi:10.1093/bioinformatics/btad198)

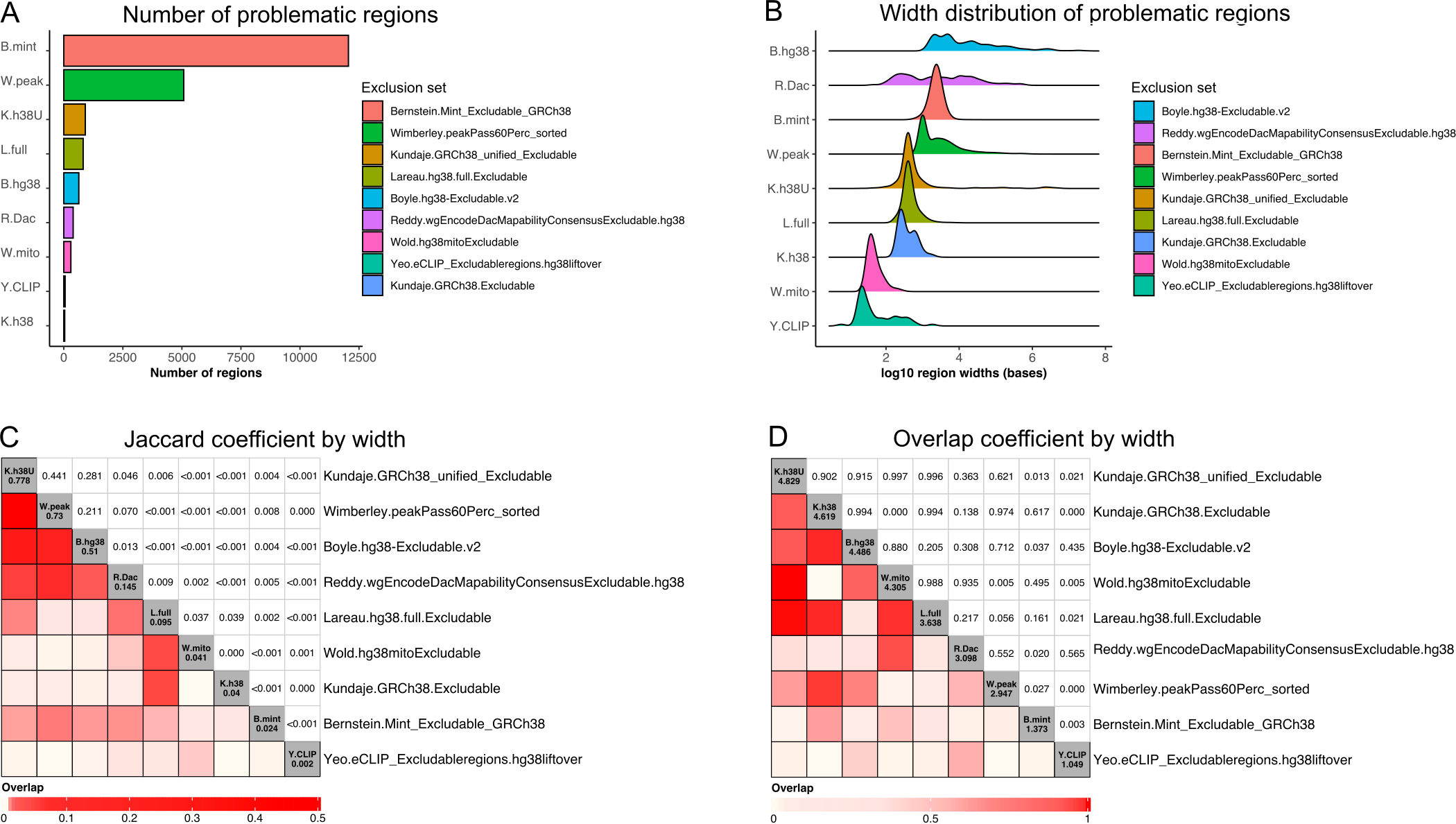

Supplement: btad198_Supplementary_Data [file btad198_supplementary_data.zip › Supplementary_Figure_S1.png]
